# Artificial intelligence–based automated computed tomography segmentation of shoulder muscles: functional muscle volume and post-operative range of motion after arthroscopic rotator cuff repair — A retrospective study

**DOI:** 10.1016/j.xrrt.2026.100892

**Published:** 2026-10-03

**Authors:** Sadaki Mitsuzawa, Akira Fujita, Hisataka Takeuchi, Koji Sakagami, Tadashi Yasuda, Shuichi Matsuda

**Affiliations:** a Department of Orthopaedic Surgery, Kyoto University Graduate School of Medicine, Kyoto, Japan; b Department of Advanced Medical Engineering and Intelligence, Kyoto University Graduate School of Medicine, Kyoto, Japan; c Department of Orthopaedic Surgery, Kobe City Medical Center General Hospital, Kobe, Japan

**Keywords:** Muscle segmentation, AI, Rotator cuff, Fatty infiltration, Computed tomography, Arthroscopic rotator cuff repair

## Abstract

**Background:**

Muscle quality and volume are important determinants of functional outcomes after arthroscopic rotator cuff repair (ARCR). Although fatty infiltration of rotator cuff muscles has been widely evaluated using semi-quantitative methods, comprehensive three-dimensional quantitative assessment of both intrinsic and extrinsic shoulder muscles remains limited. This study aimed to evaluate artificial intelligence (AI)-driven automated shoulder muscle segmentation on pre-operative computed tomography (CT) and to investigate the associations between quantitative muscle characteristics and post-operative shoulder function after ARCR.

**Methods:**

This retrospective study included 30 patients (31 shoulders) who underwent ARCR, with a mean follow-up of 20.2 months. Pre-operative CT scans were obtained using a standardized protocol with 1-mm slice thickness. Shoulder muscles were automatically segmented using an AI-based deep learning model. Six muscles—the deltoid, trapezius, subscapularis (SSc), supraspinatus, infraspinatus and teres minor complex (ISPTm), and teres major (TM)—were analyzed. Muscle composition was quantified based on Hounsfield unit thresholds and classified into functional muscle (FM), low-attenuation muscle (LAM), and adipose tissue (AT). Muscle volumes were normalized to scapular bone volume. Associations between quantitative muscle parameters and post-operative active range of motion at the final follow-up were evaluated using Pearson correlation analysis.

**Results:**

FM and AT proportions differed significantly among the 6 muscles, whereas LAM showed no significant variation. The TM demonstrated the highest FM proportion, while the supraspinatus showed the highest AT proportion. The total volumes of the SSc and TM were associated with post-operative anterior elevation (AE; r = 0.410 and 0.415, respectively), as were the FM volumes of the SSc, ISPTm, and TM (r = 0.458, 0.430, and 0.427, respectively). In contrast, neither AT nor LAM measures were significantly associated with post-operative range of motion. No muscle parameter was significantly associated with post-operative external rotation.

**Conclusion:**

AI-driven automated segmentation enabled the quantitative evaluation of shoulder muscle volume and composition on pre-operative CT. Both intrinsic and extrinsic shoulder muscles, particularly the SSc, ISPTm, and TM, were associated with post-operative AE after ARCR. Greater FM volume, rather than fatty muscle composition, was associated with better post-operative AE after ARCR. AI-based quantitative muscle assessment may provide clinically relevant information to support pre-operative evaluation.

Rotator cuff tear (RCT) is a relatively common musculoskeletal disorder. The prevalence of full-thickness RCT was reported to be 20.7% in the general population and increases with age.^16^ Arthroscopic rotator cuff repair (ARCR) is widely accepted as the gold standard treatment when the size and condition of the RCT are considered repairable. Muscle quality, including atrophy and fatty infiltration of the rotator cuff muscles, has been associated with post-operative shoulder functional outcomes and rotator cuff reparability after ARCR.^1^

Because of its simplicity and reproducibility, the Goutallier classification has been used worldwide to evaluate fatty infiltration.^3^ However, owing to the subjective and visual nature of this assessment, concerns have been raised regarding the inter-observer variability of this classification system.^10^ Recent studies focusing on fatty infiltration of shoulder muscles have shown substantial methodological advances. Evaluation techniques have evolved from qualitative to quantitative methods, from two-dimensional measurements to three-dimensional analyses, and from manual segmentation to AI-based automated segmentation.^3^, ^6^ Recent studies have demonstrated that AI-based three-dimensional musculoskeletal segmentation achieves excellent agreement with manual segmentation, with Dice similarity coefficients generally around 0.90.^6^, ^11^, ^17^ TotalSegmentator (version 2.11.0; University Hospital Zurich, Zurich, Switzerland) is an open-source, pre-trained deep learning segmentation framework that has been validated across multiple anatomical structures and has recently been applied in musculoskeletal imaging studies. More recently, studies have extended the application of TotalSegmentator to quantitative assessment of the shoulder musculature; however, these investigations have predominantly focused on patients undergoing shoulder arthroplasty.^8^, ^12^, ^14^

To date, the muscles evaluated have been mainly limited to intrinsic muscles such as the rotator cuff. Although the deltoid, trapezius, and teres major (TM) are classified as extrinsic muscles, we hypothesized that the pre-operative volume and quality of not only intrinsic but also extrinsic muscles may influence post-operative clinical outcomes after ARCR. However, these relationships have rarely been investigated.

The purpose of this study was to investigate the clinical applicability of AI-driven automated segmentation of shoulder muscles on pre-operative computed tomography (CT) scans and to examine the association between quantitative muscle characteristics and post-operative shoulder range of motion (ROM) after ARCR.

## Materials and methods

This retrospective study was performed in accordance with the principles of the Declaration of Helsinki (as revised in 2013) and approved by the Ethics Committee of our institution (approval no. 25229). Informed consent was obtained from all patients before their inclusion in the study and after publication of the anonymized results.

This study was designed as a retrospective analysis of patients with RCT who underwent ARCR at our institution between January 2022 and December 2024. The inclusion criteria were patients with RCT who were deemed suitable candidates for ARCR by 2 shoulder surgeons and who had a minimum post-operative follow-up of one year. Patients younger than 20 years of age, those with acute traumatic RCT sustained within three months of injury, and those with insufficient imaging data were excluded.

All patients underwent a standardized pre-operative imaging protocol. Within four weeks prior to ARCR, a CT scan was performed with a slice thickness of 1 mm to ensure complete visualization of the scapula. At our institution, pre-operative CT is routinely performed in patients undergoing ARCR to evaluate osseous morphology and facilitate surgical planning. CT images were imported without additional preprocessing, and analyzed using TotalSegmentator (version 2.11.0), a previously validated open-source deep learning segmentation framework with excellent agreement with manual segmentation (reported Dice similarity coefficient up to 0.943).^12^ All automated segmentation results were visually inspected by the first author prior to quantitative analysis. No segmentation failures requiring case exclusion were encountered. The deltoid, trapezius, subscapularis (SSc), supraspinatus (SSP), infraspinatus and teres minor complex (ISPTm), TM, and the scapula were identified (Fig. 1, *A* and *B*).

**Figure 1:**
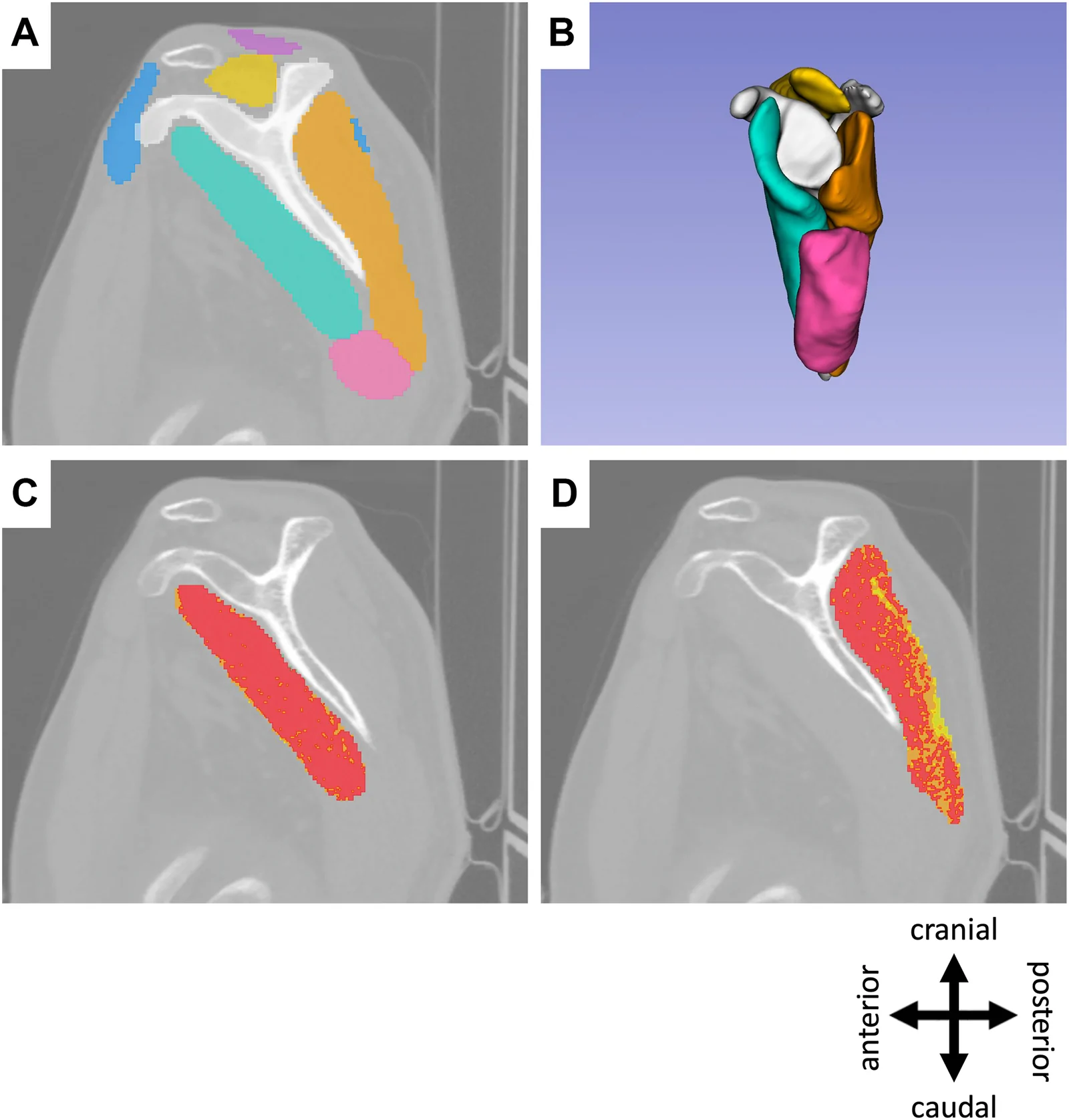
(**A**) segmented shoulder muscles and scapular bone in a sagittal slice. Blue: deltoid; purple: trapezius; green: subscapularis (SSc); yellow: supraspinatus (SSP); orange: infraspinatus and teres minor complex (ISPTm); pink: teres major (TM). (**B**) Three-dimensional reconstruction images of the shoulder muscles and scapular bone (deltoid and trapezius removed). (**C**) Distribution of muscle composition within SSc in a sagittal slice. Red: functional muscle (FM); orange: low-attenuation muscle (LAM); yellow: adipose tissue (AT). (**D**) Distribution of muscle composition within ISPTm in a sagittal slice. Red: functional muscle (FM); orange: low-attenuation muscle (LAM); yellow: adipose tissue (AT).

Following automatic segmentation, muscle phenotyping was performed for each of the 6 muscles. The regions of interest were automatically divided into 3 components based on radiodensity, as previously reported by Goodpaster et al^2^: functional muscle (FM; 30–150 HU), low-attenuation muscle (LAM; −30 to 30 HU), and adipose tissue (AT; −190 to −30 HU). The proportion of each component was calculated for each muscle (Fig. 1, *C* and *D*).

To further assess the clinical validity of the automated quantitative measurements, the relationship between the AI-derived AT percentage and the Goutallier classification for SSP was evaluated.

Total muscle volume and the volumes of each component (FM, LAM, and AT) were normalized by dividing the target volume by the volume of the scapular bone.^13^

Pre-operative active shoulder ROM was assessed before surgery. Tear size was determined based on the intra-operative arthroscopic findings and classified as none, partial, small, medium, large, or massive. Rotator cuff repair was performed using either a single-row or double-row suture anchor technique depending on the tear size and configuration.

Patients generally followed a standardized post-operative rehabilitation protocol, although minor modifications were made according to the tear characteristics. Active shoulder motion was restricted immediately after surgery, while passive ROM exercises were initiated with the patient in the supine or seated position, focusing primarily on forward elevation and external rotation (ER; limited to 30°). Restrictions on passive ER were removed at 2 weeks post-operatively. Sling immobilization was discontinued, and active elevation exercises were initiated at 4 weeks. Light work and gradual lifting were permitted after 8 weeks, and return to sports and manual labor was allowed after 12 weeks.

Post-operative active shoulder ROM, including active anterior elevation (AE) and ER, was assessed at the final follow-up visit by either the operating surgeon or a licensed physical therapist using a standard universal goniometer. Measurements were performed with the patient in the seated position according to the institutional standard protocol. The examiners who assessed post-operative ROM were blinded to the quantitative CT measurements.

Data are presented as means and standard deviations. Differences in muscle composition (FM, LAM, and AT) were analyzed using the Tukey–Kramer method. Pearson correlation coefficients (r) were calculated to assess associations between muscle composition or volume and post-operative active ROM. Correlation coefficients of 0.20–0.39, 0.40–0.59, and 0.60–1.0 were interpreted as mild, moderate, and strong correlations, respectively. Confidence intervals (CIs) for the correlation coefficients were estimated using a nonparametric bootstrap method with 2,000 resamples. Given the exploratory nature of this study and the small sample size, a post hoc power analysis was performed for the variable pair demonstrating the highest Pearson r. All statistical analyses were performed using SPSS (version 25; SPSS Corp., Armonk, NY, USA), and statistical significance was set at *P* < .05.

## Results

A total of 30 patients (31 shoulders) were included in the study. Patient demographics are summarized in Table I. The cohort consisted of 15 men and 15 women, with a mean age of 66.1 years. The mean height and body weight were 161.2 cm and 62.9 kg, respectively. The mean follow-up period was 20.2 months. Pre-operatively, the mean active ROM was 110° for AE and 41° for ER. At the final follow-up, the mean active ROM improved to 149° for AE and 58° for ER.

**Table I:** Patient demographics.

Variable	30 patients 31 shoulder
Mean age (yr)	66.1 ± 10.1
Male: female	15 : 15
Height (cm)	161.2 ± 10.5
Body weight (kg)	62.9 ± 14.7
Right: left	22 : 9
Follow-up period (months)	20.2 ± 9.2
ROM (degree)	
Pre-operative AE	110 ± 44
Pre-operative ER	41 ± 22
Post-operative AE	149 ± 26
Post-operative ER	58 ± 19

Tear type	None	Partial	Small	Medium	Large	Massive
SSP	3	6	12	7	2	1
ISP	21	1	2	6	1	0
SSc	14	2	11	3	1	0
TM	31	0	0	0	0	0

*ROM*, range of motion; *AE*, anterior elevation; *ER*, external rotation; *SSP*, supraspinatus; *ISP*, infraspinatus; *SSc*, subscapularis; *TM*, teres minor.

The volume and composition of each muscle are summarized in Table II. The total volume of the deltoid muscle was 255.2 ± 89.7 cm^3^, making it the largest muscle among the 6 evaluated. The trapezius, SSc, and ISPTm each had volumes of approximately 120–130 cm^3^. In contrast, the volumes of the SSP and TM were less than 60 cm^3^, which were smaller than those of the other muscles. The mean volume of the scapular bone was 89.5 ± 21.6 cm^3^.

**Table II:** Volume of each shoulder muscle and the scapular bone.

Muscle	Total volume	Functional muscle	Low attenuation muscle	Adipose tissue
Deltoid	255.2 ± 89.7	183.5 ± 72.7	55.2 ± 22.4	16.4 ± 8.5
Trapezius	132.7 ± 49.4	86.4 ± 37.5	30.0 ± 11.9	16.3 ± 6.5
SSc	120.3 ± 44.1	85.1 ± 37.0	29.8 ± 15.5	5.5 ± 6.3
SSP	41.3 ± 13.1	25.3 ± 11.9	10.2 ± 4.1	5.7 ± 2.8
ISPTm	133.6 ± 44.8	100.9 ± 40.7	26.3 ± 13.3	6.3 ± 3.7
TM	56.5 ± 22.4	43.5 ± 19.7	11.5 ± 8.9	1.4 ± 2.2
Scapular bone	89.5 ± 21.6			

*SSc*, subscapularis; *SSP*, supraspinatus; *ISPTm*, infraspinatus and teres minor complex; *TM*, teres major.

The normalized muscle volumes used for the correlation analyses are summarized in Table III. Mean normalized muscle volumes were 2.81 ± 0.50 for the deltoid, 1.47 ± 0.38 for the trapezius, 1.32 ± 0.24 for the SSc, 0.46 ± 0.08 for the SSP, 1.47 ± 0.24 for the ISPTm, and 0.62 ± 0.16 for the TM.

**Table III:** Normalized muscle volume of each shoulder muscle.

Muscle	Total volume	Functional muscle	Low attenuation muscle	Adipose tissue
Deltoid	2.81 ± 0.50	2.01 ± 0.44	0.62 ± 0.21	0.19 ± 0.08
Trapezius	1.47 ± 0.38	0.94 ± 0.28	0.34 ± 0.12	0.19 ± 0.07
SSc	1.32 ± 0.24	0.94 ± 0.26	0.32 ± 0.12	0.06 ± 0.06
SSP	0.46 ± 0.08	0.28 ± 0.09	0.12 ± 0.04	0.07 ± 0.03
ISPTm	1.47 ± 0.24	1.11 ± 0.27	0.29 ± 0.12	0.07 ± 0.04
TM	0.62 ± 0.16	0.48 ± 0.16	0.12 ± 0.08	0.01 ± 0.02

*SSc*, subscapularis; *SSP*, supraspinatus; *ISPTm*, infraspinatus and teres minor complex; *TM*, teres major.

Comparisons among the 6 muscles with respect to muscle composition—FM, LAM, and AT—are illustrated as percentages in Figure 2. No significant differences were observed in LAM among the muscles; however, significant differences in FM and AT were observed across various muscle combinations. The proportion of FM was highest in the TM and lowest in the SSP. Conversely, the proportion of AT was highest in the SSP and lowest in the TM.

**Figure 2:**
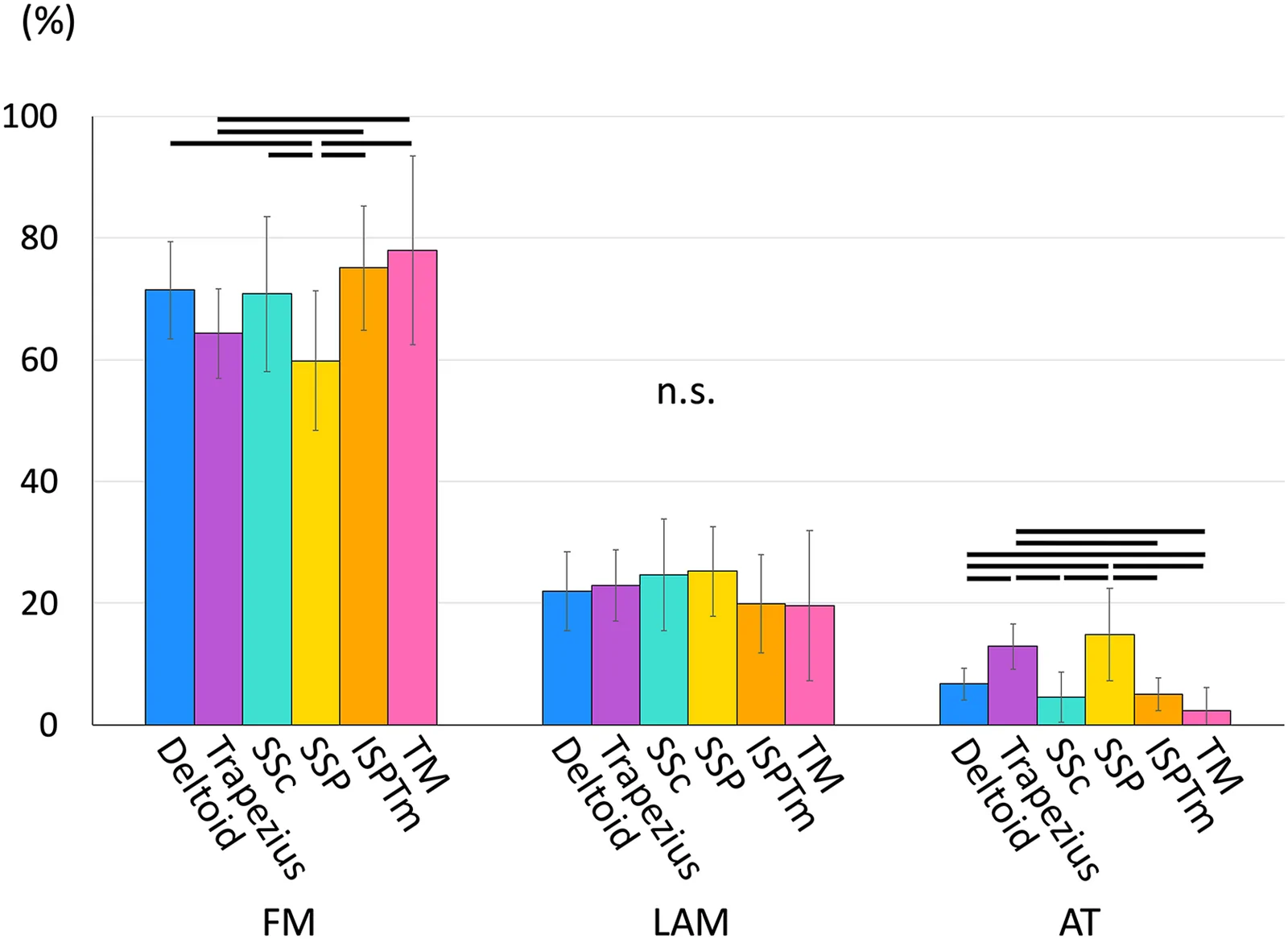
Percentage distribution of each muscle composition. Horizontal black lines above the bar graphs indicate statistically significant differences between pairs of muscle components. No significant differences were observed in LAM across the muscles; however, significant differences in FM and AT were observed in various muscle combinations. *FM*, functional muscle; *LAM*, low-attenuation muscle; *AT*, adipose tissue.

According to the Goutallier classification, 7 patients were classified as grade 1, 20 as grade 2, and 4 as grade 3; no patients were classified as grade 0 or grade 4. The Goutallier grade showed an overall positive correlation with the AT proportion of the SSP, as illustrated in Figure 3.

**Figure 3:**
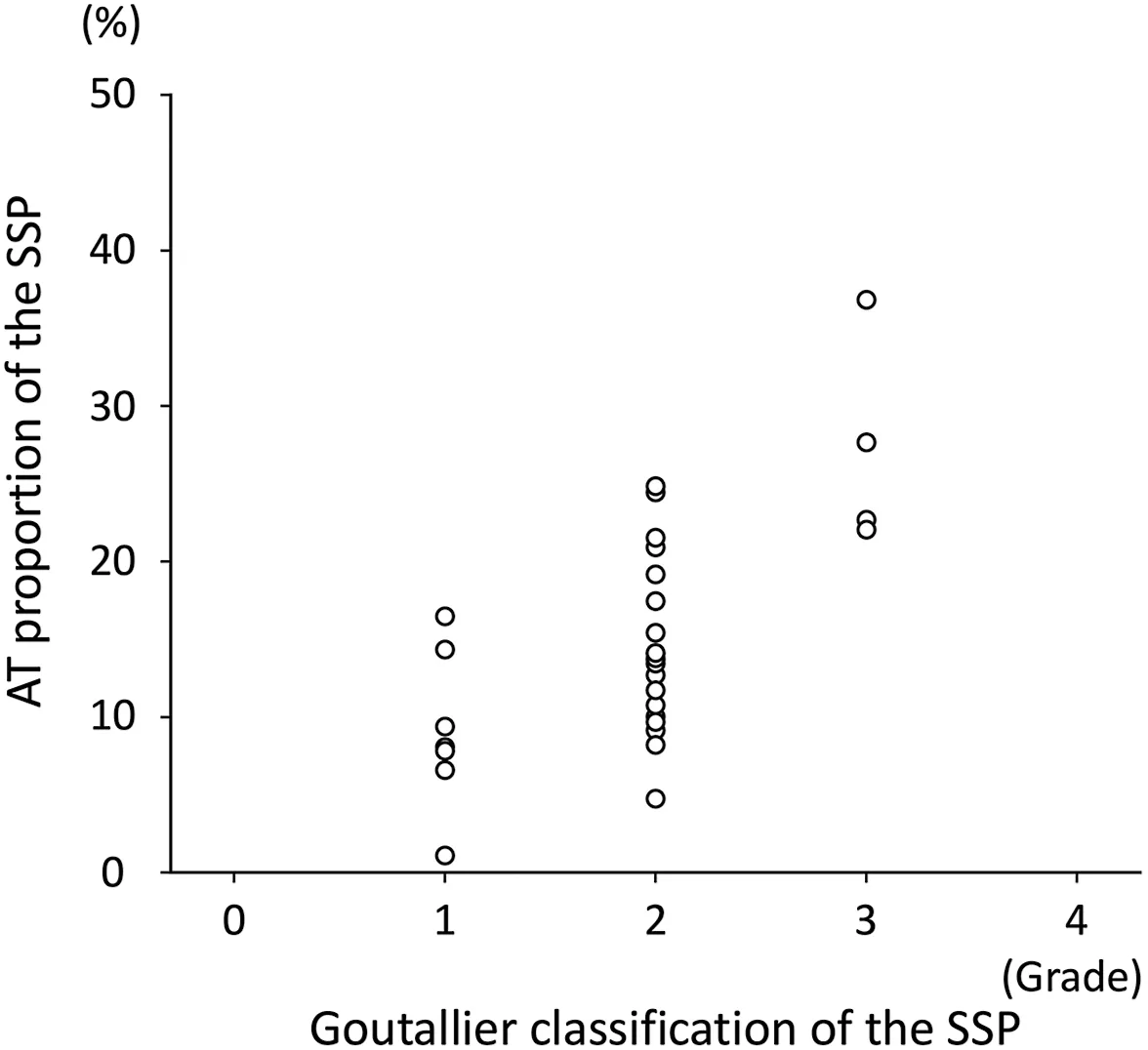
The association between the Goutallier classification and the AT proportion of the SSP. *AT*, adipose tissue; *SSP*, supraspinatus.

Associations between muscle volume and active ROM are shown in scatter plots with regression lines and 95% CIs (Tables IV and V and Fig. 4). All muscle volume measurements were normalized to the volume of the scapular bone. The total volumes of the SSc and TM were positively associated with active AE (r = 0.410, CI = 0.048∼0.627, *P* = .022 and r = 0.415, CI = −0.009∼0.675, *P* = .020, respectively). The FM volumes of the SSc, ISPTm, and TM were positively associated with active AE (r = 0.458, CI = 0.090∼0.701, *P* = .010; r = 0.430, CI = 0.146∼0.623, *P* = .016; and r = 0.427, CI = 0.045∼0.740, *P* = .017, respectively) (Fig. 4). None of the muscle components of any muscle were significantly correlated with post-operative ER. The post hoc power for detecting the observed correlation coefficient of r = 0.458 (FM volume of the SSc and active AE) with a sample size of 31 at a two-sided alpha level of 0.05 was 0.759.

**Table IV:** Each muscle composition of shoulder muscle and ROM (anterior elevation).

Muscle	Total volume	Functional muscle	Low attenuation muscle	Adipose tissue
Deltoid	r = 0.100 CI = −0.251∼0.364 *P* = .594	r = 0.211 CI = −0.149∼0.488 *P* = .256	r = −0.275 CI = −0.631∼0.190 *P* = .135	r = 0.196 CI = −0.147∼0.447 *P* = .290
Trapezius	r = 0.162 CI = −0.160∼0.419 *P* = .384	r = 0.235 CI = −0.100∼0.484 *P* = .203	r = −0.064 CI = −0.376∼0.206 *P* = .733	r = 0.061 CI = −0.381∼0.407 *P* = .746
SSc	r = 0.410 CI = 0.048∼0.627 *P* = .022	r = 0.458 CI = 0.090∼0.701 *P* = .010	r = −0.119 CI = −0.456∼0.170 *P* = .523	r = −0.117 CI = −0.515∼0.147 *P* = .530
SSP	r = 0.224 CI = −0.177∼0.559 *P* = .225	r = 0.334 CI = 0.041∼0.553 *P* = .067	r = −0.246 CI = −0.649∼0.279 *P* = .181	r = −0.014 CI = −0.434∼0.373 *P* = .940
ISPTm	r = 0.329 CI = −0.046∼0.549 *P* = .070	r = 0.430 CI = 0.146∼0.623 *P* = .016	r = −0.215 CI = −0.518∼0.102 *P* = .245	r = −0.262 CI = −0.614∼0.070 *P* = .154
TM	r = 0.415 CI = −0.009∼0.675 *P* = .020	r = 0.427 CI = 0.045∼0.740 *P* = .017	r = −0.023 CI = −0.471∼0.302 *P* = .901	r = 0.041 CI = −0.404∼0.291 *P* = .825

*ROM*, range of motion; *SSc*, subscapularis; *SSP*, supraspinatus; *ISPTm*, infraspinatus and teres minor complex; TM, teres major; *CI*, confidence interval.

**Table V:** Each muscle composition of shoulder muscle and ROM (external rotation).

Muscle	Total volume	Functional muscle	Low attenuation muscle	Adipose tissue
Deltoid	r = 0.025 CI = −0.246∼0.297 *P* = .895	r = −0.146 CI = −0.428∼0.139 *P* = .434	r = 0.280 CI = −0.059∼0.558 *P* = .127	r = 0.216 CI = −0.089∼0.471 *P* = .243
Trapezius	r = 0.082 CI = −0.234∼0.365 *P* = .662	r = 0.052 CI = −0.247∼0.353 *P* = .783	r = 0.176 CI = −0.130∼0.487 *P* = .343	r = −0.077 CI = −0.385∼0.229 *P* = .681
SSc	r = 0.043 CI = −0.270∼0.352 *P* = .817	r = 0.109 CI = −0.182∼0.429 *P* = .561	r = −0.084 CI = −0.362∼0.274 *P* = .654	r = −0.139 CI = −0.365∼0.176 *P* = .455
SSP	r = 0.316 CI = 0.078∼0.536 *P* = .083	r = 0.189 CI = −0.070∼0.449 *P* = .309	r = 0.333 CI = 0.005∼0.591 *P* = .067	r = −0.174 CI = −0.411∼0.068 *P* = .349
ISPTm	r = 0.073 CI = −0.222∼0.351 *P* = .695	r = 0.122 CI = −0.231∼0.447 *P* = .513	r = −0.066 CI = −0.424∼0.356 *P* = .726	r = −0.187 CI = −0.506∼0.189 *P* = .315
TM	r = 0.025 CI = −0.311∼0.321 *P* = .892	r = 0.060 CI = −0.215∼0.353 *P* = .750	r = −0.088 CI = −0.470∼0.261 *P* = .639	r = 0.096 CI = −0.223∼0.327 *P* = .607

*ROM*, range of motion; *SSc*, subscapularis; *SSP*, supraspinatus; *ISPTm*, infraspinatus and teres minor complex; *TM*, teres major; *CI*, confidence interval.

**Figure 4:**
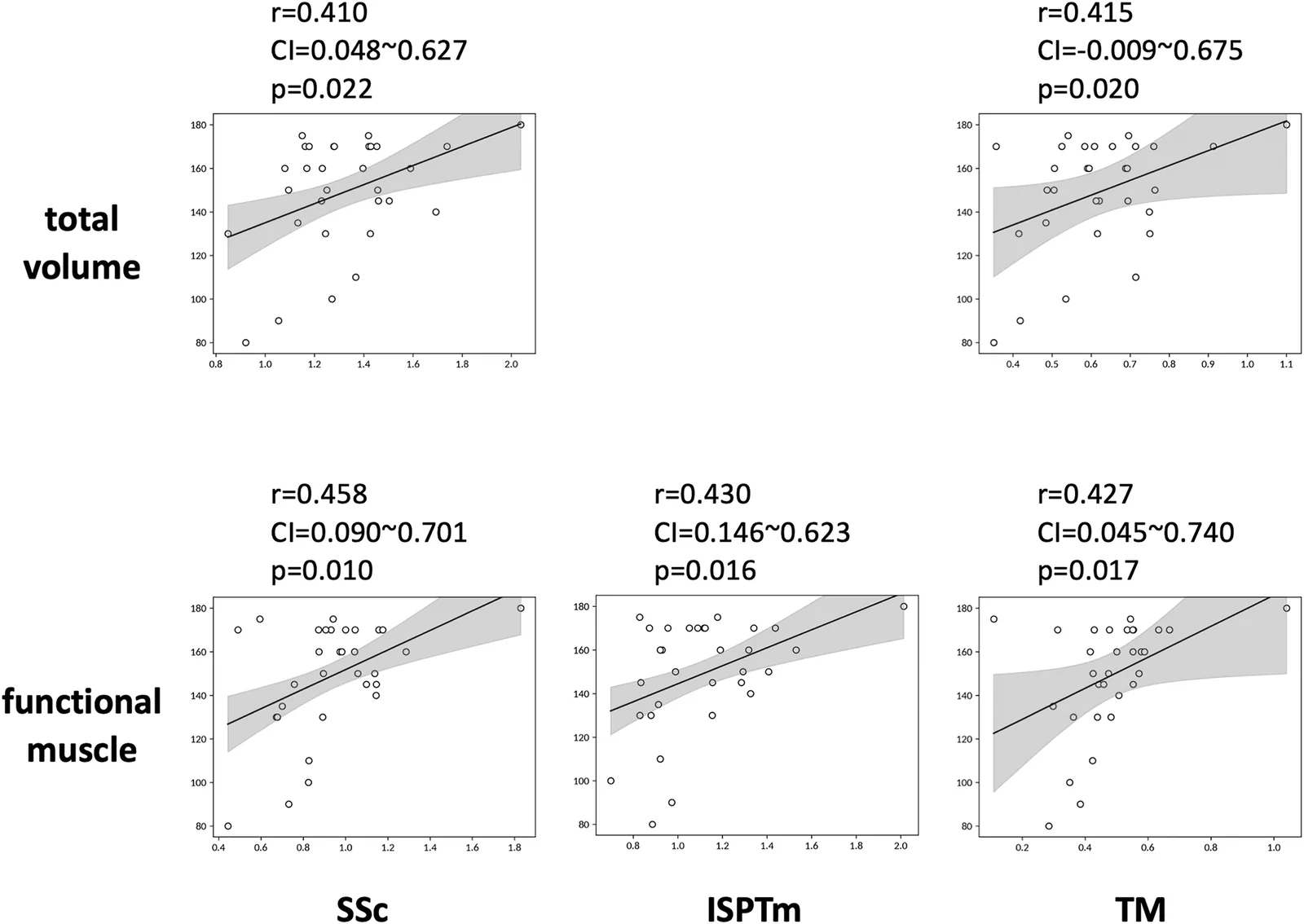
Pearson correlation analysis between anterior elevation and muscle volume. The total volume of the SSc and TM, and the FM volume of the SSc, ISPTm and TM were significantly associated with greater post-operative range of motion in anterior elevation. *SSc*, subscapularis; *FM*, functional muscle; *TM*, teres major; *ISPTm*, infraspinatus and teres minor complex.

## Discussion

An automated AI-based technique enabled the extraction of shoulder muscles from pre-operative CT images, providing quantitative metrics for evaluation in this cohort. While LAM did not show substantial variation, the proportions of FM and AT differed significantly across the 6 muscles; FM was most prominent in the TM, whereas AT was most prominent in the SSP. The total volumes of the SSc and TM, as well as the FM volumes of the SSc, ISPTm, and TM, showed moderate associations with better active AE. These findings suggest that not only intrinsic muscles but also extrinsic muscles may be associated with post-operative shoulder outcomes.

Previous studies investigating rotator cuff muscle status have predominantly relied on magnetic resonance imaging (MRI)-based assessments, often using two-dimensional, single-slice measurements or semi-quantitative grading systems.^7^ Moreover, most analyses have focused on a limited number of rotator cuff muscles, with little attention paid to non–rotator cuff muscles that may contribute to post-operative shoulder function.^15^ While recent advances in artificial intelligence have enabled automated three-dimensional muscle segmentation on CT scans, these approaches have largely been applied to patient populations undergoing shoulder arthroplasty.^8^, ^14^ To date, a comprehensive framework integrating CT-based, AI-driven three-dimensional segmentation with quantitative evaluation of muscle volume and fatty infiltration based on Hounsfield unit analysis, and linking these parameters to post-operative outcomes after ARCR, has not been well established. In this context, the present study is distinctive in that it employs pre-operative CT to perform fully automated three-dimensional segmentation. In addition, both rotator cuff muscles and extrinsic shoulder muscles, including the deltoid, trapezius, and TM, were investigated, thereby providing a more comprehensive assessment of shoulder muscle morphology and quality and their associations with post-operative outcomes.

A recent systematic review demonstrated that pre-operative fatty infiltration was associated with significantly higher re-tear rates and worse functional outcomes; however, it did not appear to reduce post-operative ROM.^18^ Although many studies have reported that post-operative outcomes are primarily associated with the SSP, more recent reports have suggested that the ISPTm complex and the SSc may play a more important role.^4^, ^5^, ^9^, ^18^ Stabilization of the glenohumeral joint and achievement of an adequate ROM require an appropriate force couple, and the balance between the SSc and the infraspinatus–teres minor complex is sometimes referred to as horizontal balance.^13^ Our findings were consistent with these reports: no significant association was observed for the SSP, whereas the SSc and ISPTm complex showed significant correlations with post-operative active ROM of AE. Interestingly, contrary to our initial hypothesis, significant associations with post-operative ROM were observed only for FM volume, whereas neither LAM nor AT demonstrated statistically significant associations with post-operative ROM.

Although numerous studies have investigated the relationship between pre-operative deltoid characteristics and post-operative ROM after reverse shoulder arthroplasty, there is a paucity of studies examining their association with post-operative ROM following ARCR. Similarly, few studies have evaluated the role of the trapezius in this context. In the present study, neither the deltoid nor the trapezius demonstrated a significant correlation with post-operative ROM after ARCR.

The TM originates from the dorsal surface of the inferior angle of the scapula and inserts onto the medial lip of the inter-tubercular groove of the humerus. Functionally, it acts as an internal rotator, adductor, and extensor of the shoulder and contributes to scapulohumeral rhythm and humeral head control. Limited evidence exists regarding the relationship between the TM and post-operative ROM after ARCR. Previous studies have primarily focused on the TM in the setting of tendon transfer procedures rather than as a potential determinant of post-operative function after rotator cuff repair. In the present study, both the total volume and FM volume of the TM demonstrated moderate correlations with post-operative AE ROM. The observed association between TM volume and post-operative ROM may reflect indirect biomechanical contributions, including glenohumeral stabilization or compensatory activation in response to residual rotator cuff dysfunction. These findings suggest that TM morphology may be associated with post-operative shoulder motion, although the underlying mechanisms remain unclear.

Several limitations warrant consideration. First, the relatively small cohort may have reduced statistical power and limited the generalizability of the findings. Although the achieved maximum power approached 0.759 for the correlation, it did not reach the conventional 0.80 threshold. Given the exploratory design, small sample size, and multiple correlations performed, there is a potential risk of type I error. The correlation analyses were unadjusted for potential confounders, including age, sex, baseline ROM, tear size, tendon involvement, repair technique, and repair integrity; multivariable analysis was not feasible given the small sample. Therefore, the findings should be interpreted with caution. Second, the minimum follow-up period was one year. Muscle atrophy, fatty infiltration, and functional recovery may continue to evolve over longer follow-up periods. Therefore, the present findings may not fully reflect long-term post-operative outcomes. Third, post-operative MRI was not routinely performed to assess repair integrity. Instead, MRI was obtained only in patients with persistent pain, decreased shoulder ROM, or suspected structural failure. Among the 30 patients, only 1 was confirmed to have a retear on post-operative MRI. Therefore, repair integrity could not be systematically evaluated as a potential confounding factor. Fourth, post-operative shoulder function was assessed only by active AE and ER. Internal rotation and validated patient-reported or clinician-based functional outcome scores were not systematically evaluated. Therefore, the relationship between AI-derived muscle characteristics and overall clinical function remains to be clarified. Fifth, muscle segmentation and fatty infiltration analyses were performed using an AI-assisted automated approach on thin-slice CT images. Although this method provides rapid and reproducible quantification, segmentation accuracy in the shoulder region may be influenced by complex anatomical structures and disease-related changes, potentially leading to measurement variability. All segmentations underwent visual quality control, and no segmentation failures requiring exclusion or manual correction were identified. Nevertheless, automated segmentations were not directly validated against expert manual segmentations in this cohort; therefore, accuracy and agreement could not be quantified, and external validation remains necessary.

## Conclusion

AI-driven automated segmentation enabled comprehensive quantitative analysis of shoulder muscle volume and composition on pre-operative CT scans. Greater total or FM volume of the SSc, ISPTm, and TM was associated with better post-operative AE. These findings support the feasibility of AI-based quantitative assessment of shoulder muscles; however, further studies with larger cohorts and validation against manual segmentation are required to determine its clinical utility in patients undergoing ARCR.

## Disclaimers:

Funding: No funding was disclosed by the authors.

Conflicts of interest: The author, their immediate family, and any research foundation with which they are affiliated have not received any financial payments or other benefits from any commercial entity related to the subject of this article.

## Declaration of generative AI and AI-assisted technologies in the writing process

Image analyses were automatically performed using TotalSegmentator (version 2.11.0), an open-source, pre-trained deep learning model.

## Availability of data and material

The datasets used and/or analyzed during the current study are available from the corresponding author upon reasonable request.
